# Gender- and Age-Specific Differences in Resting-State Functional Connectivity of the Central Autonomic Network in Adulthood

**DOI:** 10.3389/fnhum.2019.00369

**Published:** 2019-10-17

**Authors:** Jia-Hong Sie, Yin-Hua Chen, Yuo-Hsien Shiau, Woei-Chyn Chu

**Affiliations:** ^1^Department of Biomedical Engineering, National Yang-Ming University, Taipei, Taiwan; ^2^Research Center for Mind, Brain and Learning, National Chengchi University, Taipei, Taiwan; ^3^Graduate Institute of Applied Physics, National Chengchi University, Taipei, Taiwan

**Keywords:** resting-state functional magnetic resonance imaging, functional connectivity, central autonomic network, posterior mid-cingulate cortex, ventromedial prefrontal cortex, default mode network, age, gender

## Abstract

Previous functional imaging studies have identified the role of central autonomic network (CAN) in autonomic regulation during various tasks. However, its variability with respect to gender and age, particularly in the resting state, remains poorly understood. Therefore, in this study we systematically investigated gender- and age-related differences in the resting-state functional connectivity (rsFC) seeded from core regions of this network, namely posterior mid-cingulate gyrus (pMCC), left amygdala, right anterior and left posterior insula, and ventromedial prefrontal cortex (vmPFC), using a large cross-sectional adulthood sample. Results revealed that each of the seeded connectivity maps engaged in at least one of the large-scale brain networks including sensorimotor, attentional, basal ganglia, limbic, and default mode networks (DMN). In the early-adulthood stage, females had stronger negative rsFC in pMCC and right anterior INS (aINS) with the medial DMN than males, possibly reflecting their greater suppression of the sympathoexcitation associated with sex hormonal estrogen. Whereas in the late-adulthood stage, they showed stronger positive rsFC in pMCC with postcentral gyrus and weaker negative rsFC with the most DMN, possibly relating to their higher risk of depression, anxiety, and dementia than males after menopause. Moreover, females demonstrated reduced negative rsFC in pMCC with dorsal PCUN/PCC and left AG with advancing age, whereas males showed the opposite pattern, namely increased positive rsFC, in pMCC with right SMG, and in vmPFC with ventral PCUN. We interpret these results as their differences of altered autonomic regulation associated with pain experience and reflective movement, respectively, due to aging. In sum, our findings add in literature that autonomic responses can be also represented intrinsically in the resting brain, and gender- and age-related differences might be associated with sex hormones and sensorimotor abilities, respectively.

## Introduction

The central autonomic network (CAN) plays a crucial role in the regulation of central and autonomic nervous system (ANS) such as the control of body visceral functions, maintenance of homeostasis, and adaptation to internal or external challenges (Benarroch, 1993; Thayer and Lane, 2000; Hagemann et al., 2003). It was first characterized in experimental animals and then also demonstrated in humans (mainly adults) with the advent of brain-imaging methods (Benarroch, 1993; Verberne and Owens, 1998; Saper, 2002; Beissner et al., 2013; Critchley et al., 2013; Macey et al., 2015). The anterior cingulate (ACC) and midcingulate cortices (MCC), insula (INS), ventromedial prefrontal cortex (vmPFC), hypothalamus, mediodorsal thalamus, amygdala (AMYG), and hippocampal function (HF) have been identified as component regulatory regions of this network by a meta-analysis (Beissner et al., 2013). Among them, four regions, namely posterior midcingulate cortex (pMCC), left AMYG, right anterior INS (aINS), and left posterior INS (pINS), have been found to consistently engage in autonomic regulation across cognitive, affective, and somatosensory-motor tasks (Beissner et al., 2013). Specifically, the pMCC mediates context-driven modulation of cardiac function via sympathetic output (Critchley et al., 2000, 2003; Goswami et al., 2011). The AMYG modules both sympathetic and parasympathetic regulation reflecting the need for balancing increased sympathetic and decreased parasympathetic outflow (Critchley et al., 2000; Williams et al., 2004; Kimmerly et al., 2005; Napadow et al., 2008). The right aINS is related to the limbic system, with the ventral part associated with sympathetic regulation and dorsal part associated with parasympathetic regulation (Critchley et al., 2000; Nagai et al., 2004a; Kimmerly et al., 2005; Wong et al., 2007b; Goswami et al., 2011; Maihofner et al., 2011; Napadow et al., 2013). The left pINS is related to sensorimotor integration, and generally engages sympathetic modulation (Williams et al., 2004; Napadow et al., 2008; Goswami et al., 2011; Napadow et al., 2013). In other word, the pMCC and the left pINS are involved in sympathetic regulatory function, and the left AMYG and the right aINS are involved in both sympathetic and parasympathetic regulatory functions.

The CAN regions, based on the dichotomy of sympathetic and parasympathetic divisions of the ANS, also show a close relationship with the large-scale brain networks (Beissner et al., 2013). That is, the sympathetic-associated CAN regions (e.g., lateral PFC, aINS, MCC, and superior parietal lobule) were found to predominate in the so-called task-positive networks such as central executive and salience networks, whereas the parasympathetic-associated CAN regions [e.g., precuneus (PCUN), posterior cingulate cortex (PCC), inferior parietal lobule, and HF] predominate in the task-negative network, that is, default mode network (DMN) (Beissner et al., 2013). Notably, the vmPFC, often considered as a DMN region, indeed shows parasympathetic regulation (e.g., cardiac vagal tone) (Critchley et al., 2000; Wong et al., 2007b; Ziegler et al., 2009; Goswami et al., 2011). Moreover, it also shows sympathetic regulation as a significant component of a limbic network (Kimmerly et al., 2007; Beissner et al., 2012; Zhang et al., 2014). Therefore, the four regions mediating autonomic activity across tasks, namely pMCC, left AMYG, right aINS, and left pINS, as well as vmPFC, and their relationship with the large-brain networks from the perspective of sympathetic and parasympathetic regulation, were considered of great importance in the CAN.

It has been found that males and females differ in their autonomic responses that are associated with brain activation or deactivations within CAN regions (Kimmerly et al., 2007; Wong et al., 2007a; Macey et al., 2015, 2016). For example, during the handgrip exercise and Valsalva maneuver, males had larger heart rate responses and stronger cortical activations in the right INS and dorsal ACC than females (Kimmerly et al., 2007; Wong et al., 2007a; Macey et al., 2016). Whereas greater deactivations in the ventral ACC and vmPFC were additionally found in males compared to females when they had lower body negative pressure (Kimmerly et al., 2007). The authors interpreted the greater changes in men compared to women due to the more active autonomic responses to the maneuvers and suggested that the sympathoexcitation in women may be attenuated by sex hormonal estrogen (Wong et al., 2007a; Macey et al., 2016). Such findings imply that males and females might be different particularly in certain periods of adulthood such as menopause. By now, gender- and age-related differences of autonomic regulation in adults when measuring heart rate variability in electrocardiography have been widely investigated (Umetani et al., 1998; Kuo et al., 1999; Bonnemeier et al., 2003; Almeida-Santos et al., 2016). A general consensus is that females and males have their own dominant parasympathetic and sympathetic regulations when they were young and these regulations gradually decrease with advancing age due to a significant decrease of nocturnal parasympathetic activity. Hence, the preservation of parasympathetic function may serve as a biomarker relating to the healthy longevity and vitality in late life span (Zulfiqar et al., 2010). Specifically, for women, an important period in adulthood is the menopause. Premenopausal women have stronger parasympathetic regulation by showing a greater high-frequency power than postmenopausal ones (Liu et al., 2003). Moreover, they have dominant vagal and subordinate sympathetic activity compared with age-matched men (Kuo et al., 1999), but such gender-related differences were not detected in postmenopausal women compared with age-matched men (Kuo et al., 1999; Liu et al., 2003). However, gender-related differences in different stages of adulthood at the neural level associated with CAN remain poorly understood by far (Macey et al., 2015).

From the clinical perspective, a model of neurovisceral integration in emotion regulation and dysregulation has been proposed to explain a relative reduction in vagally mediated heart rate variability in major depressive and generalized anxiety disorders (Thayer and Lane, 2000; Hagemann et al., 2003; Nugent et al., 2011). Moreover, previous functional magnetic resonance imaging (fMRI) studies indicated that obstructive sleep apnea patients had altered signals appeared in the CAN regions, namely left aINS, bilateral putamen, and posterior ventral thalamus, with earlier heart rate increases during inspiratory loading (Macey et al., 2006). An observational study also showed that mild cognitive impairment participants had significant parasympathetic deficits in the Ewing’s Battery of standardized bedside cardiovascular reflex tests and reduction in the high frequency domain compared with controls (Collins et al., 2012). In the elderly, neurodegenerative conditions such as Parkinson’s disease and multiple system atrophy are also found to be associated with autonomic dysfunction (Buijs and Swaab, 2013; Sklerov et al., 2018). Specifically, Parkinson’s disease patients with higher burden of autonomic symptoms show reduced hypothalamic functional connectivity than those with lower burden of the symptoms, suggesting the involvement of CAN (Dayan et al., 2018). The autonomic dysfunction also occurs in four types of dementia, particularly in patients with Parkinson’s disease dementia and dementia with Lewy bodies (Allan et al., 2007). Although clinical studies of CAN dysfunction are not yet clear, they consistently implicate use of fMRI as a promising biomarker of neurodegenerative disease.

Other than the aforementioned functional imaging findings, investigations on the functionally connected networks but during task-free (i.e., resting state) conditions, referred to resting-state functional connectivity (rsFC), can provide insight into the dynamic, ongoing, and intrinsic property of brain networks (Fox et al., 2005; Yeo et al., 2011). It has been shown that the large-scale brain networks at the resting state are different in males compared to females and they are plastic across the lifespan (Biswal et al., 2010; Scheinost et al., 2015). For instance, female adults have stronger rsFC strength within the DMN compared to male ones, whereas male ones are more active in the sensorimotor network (Filippi et al., 2013). Young adults (including males and females) demonstrate stronger reciprocal couplings between the central executive network and DMN via salience network than the middle-aged or the elderly ones, demonstrating the changes in cognitive functions due to aging (He et al., 2013; Cao et al., 2014; Geerligs et al., 2015; Marstaller et al., 2015). In terms of CAN rsFC, we have used the CAN core regions as seeds to examine the connectivity in young male adults with different levels of sporting experience (Sie et al., 2019). Results confirmed the close relationship between the CAN and large-brain networks in sensory, motor, and cognitive domains. Particularly, adults with greater sporting experience demonstrated enhanced connectivity between pMCC and right supramarginal gyrus (SMG), between right aINS and dorsal ACC, and between left AMYG and right putamen, compared to adults without any related experience (Sie et al., 2019). We interpreted the results as a stronger interregional coupling in sensorimotor and cognitive control, and in motor skill consolidation, respectively. However, the findings were limited in young males only (from 18 to 25 years old). The specific differences in rsFC of the CAN with respect to age and gender have not been systematically investigated.

To address the aforementioned issues, the present study investigated gender- and age-specific differences in rsFC of the CAN in males and females at different stages of adulthood using a large cross-sectional adulthood sample from the Southwest University adult lifespan database [Wei et al. (2018); 307/187 females/males; age range = 19–80 years]. The dataset was segregated by gender and divided into early-, middle-, and late-adulthood stages in females and males, where menopause would be approximately in the middle-adulthood of females. We used a seed-based correlation analysis in the five core regions (i.e., pMCC, left AMYG, right aINS, left pINS, and vmPFC) to explore their whole-brain rsFC. Hypotheses were formulated based on the current knowledge about gender- and age-related differences in autonomic processing, and the corresponding findings in task-based and task-free imaging studies associated with the characteristics of the seeds (i.e., sympathetic/task-positive or parasympathetic/task-negative regulation). Specifically, premenopausal females would show stronger rsFC with the DMN that are associated with parasympathetic regulation compared to males at the same age, whereas males at the same period of adulthood would show stronger rsFC associated with sympathetic regulation. These differences would disappear in the mildlife adults as postmenopausal females have weaker parasympathetic and comparatively stronger sympathetic regulations after menopause. For the effect of age, the rsFC strength of all seeds would generally decrease as a function of age to reflect the weaker autonomic regulatory function due to aging, which might be clinically associated with neurodegenerative diseases.

## Materials and Methods

### Participants and MRI Acquisition

We used the Southwest University adult lifespan dataset in a large cross-sectional population-based sample [Wei et al. (2018); 307/187 females/males; age range = 19–80 years]. All participants were healthy and free of psychiatric disorders, neurological disorders, and had no history of head trauma. The dataset collection was performed in compliance with the ethical principles of the Declaration of Helsinki and was approved by the Research Ethics Committee of the Brain Imaging Center of Southwest University. Before the experiment, each participant gave a written informed consent to data collection. Two participants were excluded because one (Sub-031397) had no functional image and the other one (Sub-031334) had fewer functional volumes than the others. The remaining 492 participants (305 females, 187 males, age from 19 to 80 years) were divided at the age of 35 and 60 year into six groups, including 109 early-adulthood (19–34 years) females (EF) and 72 males (EM), 127 middle-adulthood (35–59 years) females (MF) and 64 males (MM), and 69 late-adulthood (60–80 years) females (LF) and 51 males (LM).

Participants received instructions to lie down, close their eyes, and rest without thinking about any specific thing but to refrain from falling asleep. Images were collected on a Siemens Trio 3.0T MRI scanner (Erlangen, Germany). Resting-state fMRI scans were collected using gradient echo echo-planar imaging (GRE-EPI) sequences with repetition time (TR)/echo time (TE) of 2000/30 ms, a 90° flip angle (FA), and slice thickness/slice gap of 3/1 mm. The acquisition matrix was 64 × 64, with a 220 mm × 220 mm field of view (FOV). Each scan session was 484 s long and comprised 242 functional volumes, with each volume consisting of 32 axial slices. For spatial normalization and localization, T1-weighted images were acquired using the following magnetization-prepared rapid gradient echo (MPRAGE) sequence: TR/TE = 1900/2.52 ms, inversion time (TI) = 900 ms, FA = 90°, FOV = 256 mm × 256 mm, voxel size = 1.0 mm × 1.0 mm × 1.0 mm, number of slices = 176.

### Image Preprocessing

Image preprocessing was carried out using Data Processing Assistant for rs-fMRI (DPARSF) in Data Processing and Analysis for Brain Imaging toolbox (DPABI) (Yan et al., 2016) based on Statistical Parametric Mapping (SPM12^1^) performed in Matlab R2016b (The Math Works Inc.). The preprocessing included the following steps: (1) the first 10 volumes of each participant were discarded, (2) slice timing correction for timing offsets using since interpolation at the middle time point of TR, and (3) head motion correction using a six-parameter spatial transformation. Six EF, five EM, two MF, four MM, four LF, and four LM participants were excluded by the criterion with head motion >3.0 mm or 3.0° of head rotation. To reduce nuisance covariates from head motion, white matter and cerebrospinal fluid, (4) the functional data were then processed within nuisance covariate regressions including (i) a Friston 24-parameter model (Friston et al., 1996), (ii) a scrubbing model (spike regression) identifying each “bad” time points setting a threshold of frame-wise displacement (FD) > 0.5 mm as well as one back and two forward neighbors as a separate regressor at the individual level (Satterthwaite et al., 2013; Yan et al., 2013). Moreover, to avoid the significant impacts on negative correlations introduced by global signal regression (GSR) (Murphy et al., 2009; Saad et al., 2012), instead we adopted an anatomical component base noise reduction method (aCompCor) (Behzadi et al., 2007; Chai et al., 2012). The aCompCor has another advantage of good assessment of physiological fluctuations for the reduction of noise in blood oxygenation level-dependent (BOLD) fMRI data. It was used to extract the first five principal components from a combined white matter/cerebrospinal fluid mask by setting a probability threshold 0.99 on one’s own tissue segmentation maps (Behzadi et al., 2007; Chai et al., 2012). Next, the individual structural images were (5) co-registered to the resulting functional data for each participant and (6) subsequently spatially normalized to Montreal Neurological Institute (MNI) space by using the diffeomorphic anatomical registration through exponentiated lie algebra (DARTEL) segmentation (Ashburner, 2007) and resampled to 3 mm isotropic voxels and (7) a Gaussian kernel of 6 mm full width at half maximum for spatial smoothing. Finally, (8) the temporal band-pass filtering (0.01–0.1 Hz) was carried to reduce low-frequency drift and high-frequency physiological aliasing.

### Seed-Based Correlation and Group Analyses

The seed of pMCC, left AMYG, right aINS, left pINS, and vmPFC was a sphere with 6 mm radius peaked at (4, 0, 48), (−22, −8, −16), (34, 20, 4), (−32, −18, 12), and (−4, 38, −16) in MNI space, respectively, as suggested by previous studies (Ziegler et al., 2009; Beissner et al., 2013). The time course of each seed was correlated with time course of all voxels within the whole-brain mask from the SPM’s *a priori* brain for each participant. The strength of rsFC was defined as Fisher transformed correlation coefficients (i.e., *z*-values) for improving the normality. Individual functional connectivity maps for each group underwent a two-tailed one-sample *t*-test (compared with zero) to determine significant positive and negative correlations with the seeds voxel by voxel. As a large sample size allows a stringent threshold for multiple comparison correction (Tian et al., 2007), we chose a *p* < 0.05 permutation test (PT) by setting the number of permutations at 5,000 with a voxel-wise threshold of two-tailed *z* > 4.18 (*p* < 0.0001) for each group, as implemented in permutation analysis of general linear models (Winkler et al., 2016) and integrated into the DPABI. The surface visualizations were illustrated using BrainNet Viewer (Xia et al., 2013). The union set of the resultant 3D positive and negative connectivity maps among the six groups was used as masks for subsequent group analyses, referring to pMCC(+), pMCC(−), left AMYG(+), left AMYG(−), right aINS(+), right aINS(−), left pINS(+), left pINS(−), vmPFC(+), and vmPFC(−) network maps, respectively.

For testing the gender-related differences in the positive and negative networks in the early-, middle-, and late-adulthood stages, respectively, 30 separate two-tailed two-sample *t*-tests were used. A PT correction (*p* < 0.05) by setting the number of permutations as 5,000 with a voxel-wise threshold of two-tailed *z* > 2.58 (*p* < 0.005) was used and the individual *z*-values from that regions showing significant gender differences were extracted for illustration. Next, five separate one-way analyses of covariance (ANCOVAs) were used to examine the effect of age in the positive and negative networks in the male and female groups, respectively. A PT correction (*p* < 0.05) by setting the number of permutations as 5,000 with a voxel-wise threshold of two-tailed *z* > 2.33 (*p* < 0.01) was used for the ANCOVAs. Then, the individual *z*-values from clusters that showed a significant effect of age were extracted and compared with Bonferroni correction as *post hoc* analyses using Statistical Package for the Social Sciences 20.0 (SPSS, Chicago, IL, United States).

## Results

### Demographic Characteristics of Participants

Demographics of the participants were shown in the Supplementary Information. The head motion (i.e., mean FD) derived from Power’s relative root mean square algorithm (Power et al., 2012) significantly increased as a function of age in both females and males whereas the gray matter volume (GMV) of the whole brain significantly decreased as a function of age (Supplementary Tables S1, S2). On the other hand, there were no significant differences in the head motion (Supplementary Figure S1A) between males and females in different stages of adulthood, but males consistently showed greater GMV than females in the early- (*p* < 0.001), middle- (*p* < 0.01), and late-adulthood (*p* < 0.001) stages (Supplementary Figure S1B). Therefore, the FD and GMV were both included as covariates in the ANCOVAs and two-sample *t*-tests to attenuate the effect of head motion and cortical atrophy (Cao et al., 2014).

### General Pattern of Positive and Negative Seeded Connectivity Maps in Different Groups

**FIGURE 1:**
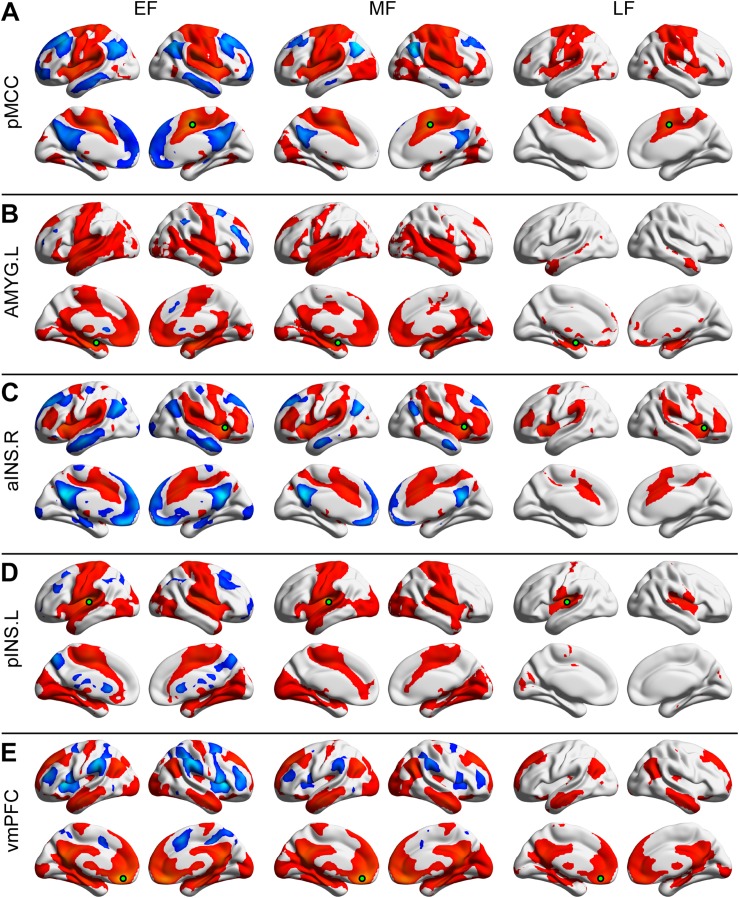
Positive and negative functional connectivity maps seeded from the posterior midcingulate cortex (pMCC) **(A)**, the left amygdala (AMYG) **(B)**, the right anterior insula (aINS) **(C)**, the left posterior insula (pINS) **(D)**, and the ventromedial prefrontal cortex (vmPFC) **(E)** for the early- (EF, left column), middle- (MF, middle column), and late-adulthood females (LF, right column) (the seed was a 6 mm radius sphere shown in green; red and blue color indicated positive and negative connectivity maps, respectively, with a permutation test correction threshold of *p* < 0.05).

**FIGURE 2:**
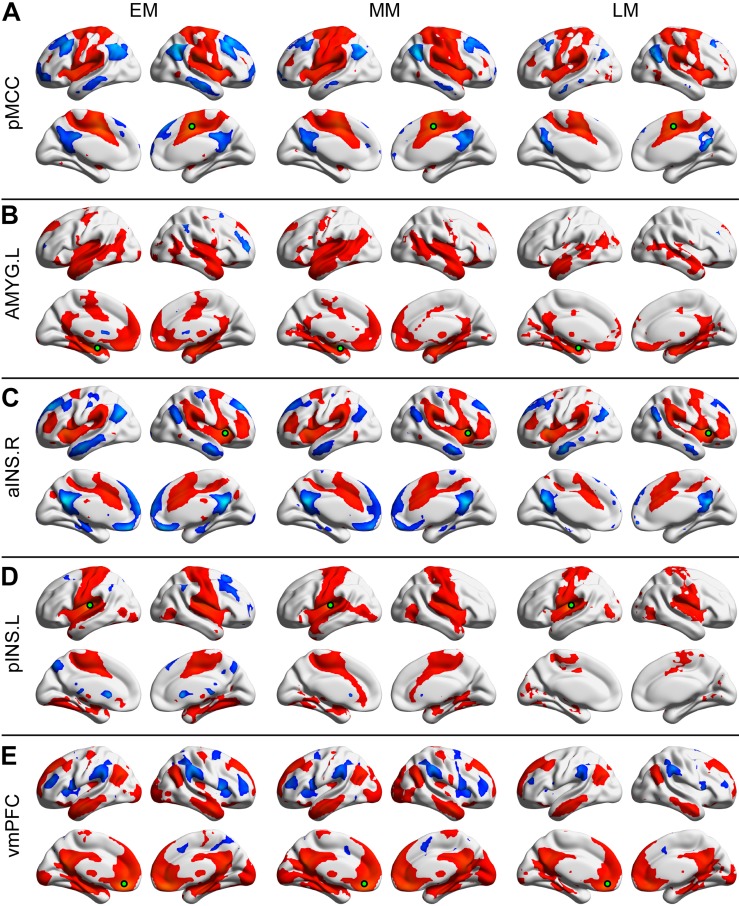
Positive and negative functional connectivity maps seeded from the posterior midcingulate cortex (pMCC) **(A)**, the left amygdala (AMYG) **(B)**, the right anterior insula (aINS) **(C)**, the left posterior insula (pINS) **(D)**, and the ventromedial prefrontal cortex (vmPFC) **(E)** for the early- (EM, left column), middle- (MM, middle column), and late-adulthood males (LM, right column) (the seed was a 6 mm radius sphere shown in green; red and blue color indicated positive and negative connectivity maps, respectively, with a permutation test correction threshold of *p* < 0.05).

Each of the seeded connectivity maps engaged in at least one of the large-scale brain networks including sensorimotor, basal ganglia, DMN, and attentional networks (Figures 1, 2). For example, as shown in the pMCC(+) network maps (Figures 1A, 2A, red color), pMCC was positively correlated with bilateral supplementary motor area, sensorimotor cortex, operculum of both frontal and parietal cortices, putamen, pallidum, and ventral thalamus, which could be associated with sensorimotor and basal ganglia networks. For the pMCC(−) network map, the regions within the DMN including PCUN, PCC, angular gyrus (AG), posterior PFC, and middle temporal gyrus were involved, except in the LF (Figures 1A, 2A, blue color). For the left AMYG(+) network maps, the regions within the limbic and basal ganglia networks including HF, vmPFC, temporal pole, putamen, pallidum, and ventral thalamus were involved, except in the LF (Figures 1B, 2B, red color). On the other hand, the left AMYG(−) networks were only detected in the lateral PFC in the EF and EM (Figures 1B, 2B, blue color). The right aINS positively correlated with the anterior MCC, SMG, premotor cortex, lateral PFC, putamen, pallidum, and ventral thalamus, as the right aINS(+) network maps, which greatly overlapped with the ventral attention and basal ganglia networks (Figures 1C, 2C, red color). The right aINS(−) network maps were similar to the pMCC(−) network maps, with the regions within the DMN including PCUN, PCC, AG, posterior PFC, and middle temporal gyrus being involved, except in the LF (Figures 1C, 2C, blue color). The left pINS(+) network maps included sensorimotor cortex, supplementary motor area, and visual cortex except in the LF, which was largely overlapped with the pMCC(+) network maps (Figures 1D, 2D, red color). Likewise, the pINS(−) network, as the pMCC(−) network, connected to the caudate, PCUN, and posterior PFC, but only in the EF and EM (Figures 1D, 2D, blue color). Finally, the vmPFC(+) network maps included the medial PFC, PCUN, PCC, AG, putamen, caudate, ventral thalamus, AMYG, HF, and middle temporal gyrus, which could be greatly associated with DMN, basal ganglia, and limbic networks (Figures 1E, 2E, red color). The vmPFC(−) network maps were detected in the bilateral aINS, dorsolateral PFC, and SMG in all groups, except in the LF (Figures 1E, 2E, blue color).

### Gender-Related Differences in the Early-, Middle-, and Late-Adulthood Stages

**FIGURE 3:**
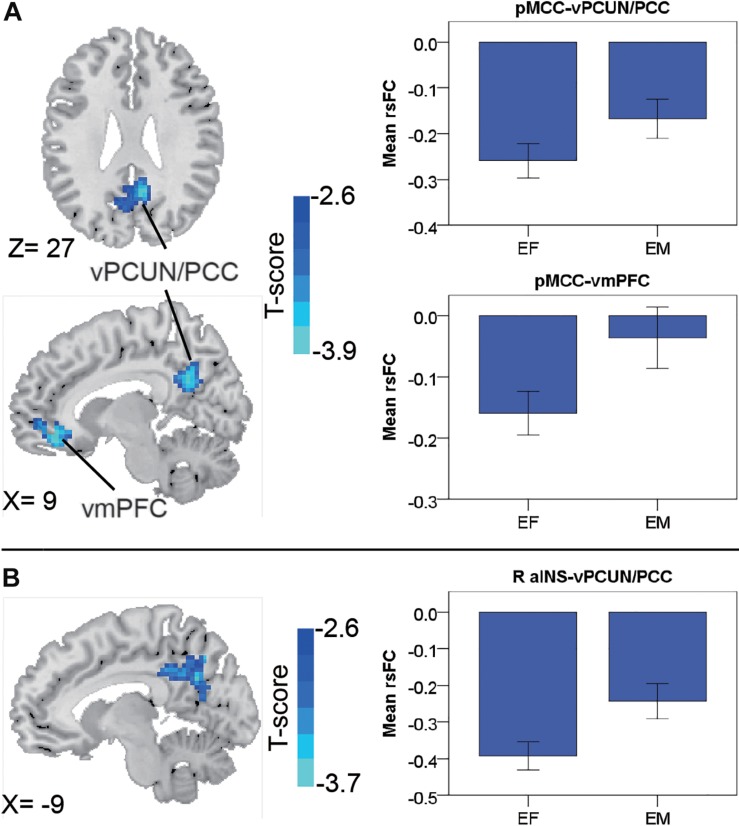
Regions showing the gender differences in the early-adulthood stage detected by two-sample *t*-test (with a permutation test correction threshold of *p* < 0.05) in the pMCC(–) network **(A)** and the right aINS(–) network **(B)**, each with the corresponding mean strength between the seed and region for the males and females shown in the bar plot, error bars indicated two standard errors (pMCC, posterior midcingulate cortex; aINS, anterior insula; vPCUN, ventral precuneus; PCC, posterior cingulate cortex; vmPFC, ventromedial prefrontal cortex; R, right).

In the early-adulthood stage, for the pMCC(+) network, no significant gender-related differences were found; whereas for the pMCC(−) network in the same stage, females showed stronger anti-correlations with the ventral PCUN/PCC and right vmPFC compared to males (i.e., EF < EM in case of negative values; Figure 3A and Table 1). Differences in similar regions (i.e., the ventral PCUN/PCC), with a similar trend, were also found in the right aINS(−) network (i.e., EF < EM in case of negative values; Figure 3B and Table 1). There were no gender-related differences in this stage (i.e., early adulthood) for all of the other seeded-networks.

**TABLE 1:** Regions showing the gender differences in different stages of adulthood.

**Region (BA)**		**MNI coordinates**			**Peak value (*F*-score)**	**Cluster size (voxels)**
		***x***	***y***	***z***		
**Early-adulthood (EF vs. EM)**						
pMCC(−): vPCUN/PCC (31/7)	B	9	–54	27	–3.85	390
pMCC(−): vmPFC (10)	R	9	42	–15	–3.77	154
R aINS(−): vPCUN/PCC (31/7)	B	–9	–60	30	–3.65	252
**Middle-adulthood (MF vs. MM)**						
No significant differences						
**Late-adulthood (LF vs. LM)**						
pMCC(+): POST (3)	R	30	–33	60	3.50	218
pMCC(−): PCUN/L AG/L MT (7/31/39/19)		0	–57	45	3.71	881
pMCC(−): FEF (8)	B	–6	27	57	3.66	345
pMCC(−): Cerebellum Crus I	B	12	–75	–30	3.71	339
L AMYG(+): Cerebellum VI/Crus I	L	–21	–72	–33	3.60	216
L pINS(−): Cerebellum Crus I	B	33	–78	–24	3.58	192
vmPFC(−): SMG (40)	R	60	–45	36	4.23	310
vmPFC(−): dlPFC (46)	R	27	42	21	4.15	151
vmPFC(−): SMA (6)	R	18	15	45	3.32	115

*Thresholds were set at *p* < 0.05, permutation test corrected. BA, Brodmann area; MNI, Montreal Neurological Institute; EF, early-adulthood females; EM, early-adulthood males; MF, middle-adulthood females; MM, middle-adulthood males; LF, late-adulthood females; LM, late-adulthood males; pMCC, posterior midcingulate cortex; aINS, anterior insula; AMYG, amygdala; vmPFC, ventromedial prefrontal cortex; vPCUN, ventral precuneus; PCC, posterior cingulate cortex; POST, postcentral gyrus; AG, angular gyrus; MT, middle temporal visual area; FEF, frontal eye field; SMG, supramarginal gyrus; dlPFC, dorsolateral prefrontal cortex; SMA, supplementary motor area; L, left; R, right; B, bilateral.*

In the middle-adulthood stage, no significant gender-related differences were found for all the seeded-networks.

**FIGURE 4:**
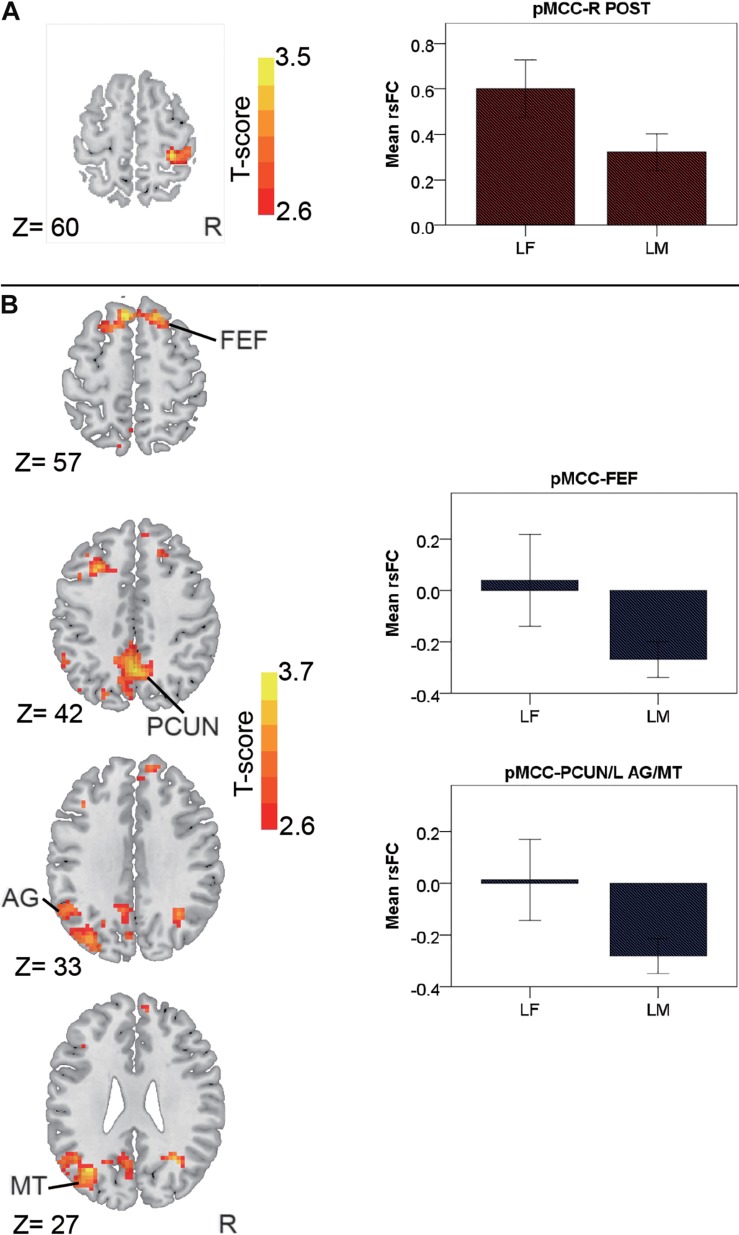
Regions showing the gender differences in the late-adulthood stage detected by two-sample *t*-test (with a permutation test correction threshold of *p* < 0.05) in the pMCC(+) network **(A)** and the pMCC(-) network **(B)**, each with the corresponding mean strength between the seed and region for the males and females shown in the bar plot, error bars indicated two standard errors (pMCC, posterior midcingulate cortex; POST, postcentral gyrus; FEF, frontal eye field; PCUN, precuneus; AG, angular gyrus; MT, middle temporal visual area; L, left; R, right).

**FIGURE 5:**
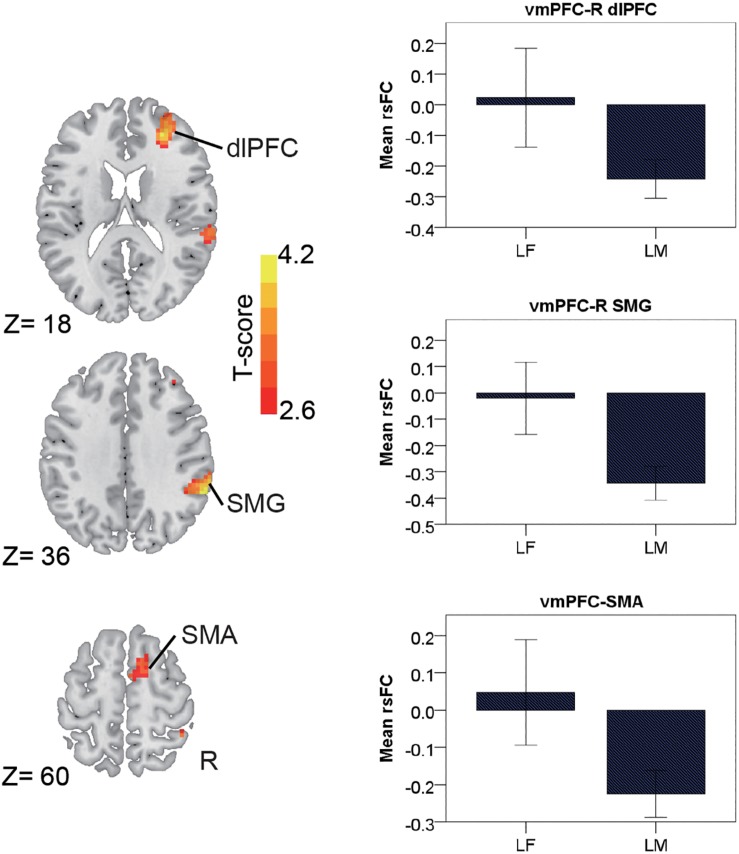
Regions showing the gender differences in the late-adulthood stage detected by two-sample *t*-test (with a permutation test correction threshold of *p* < 0.05) in the vmPFC(–) network, each with the corresponding mean strength between the seed and region for the males and females shown in the bar plot, error bars indicated two standard errors (vmPFC, ventromedial prefrontal cortex; dlPFC, dorsolateral prefrontal cortex; SMG, supramarginal gyrus; SMA, supplementary motor area; R, right).

In the late-adulthood stage, for the pMCC(+) network, females showed stronger connectivity with the right postcentral gyrus than males (i.e., LF > LM; Figure 4A and Table 1). By contrast, for the pMCC(−) network, males showed stronger strength of connectivity with the bilateral frontal eye field, PCUN, left AG, and middle temporal visual area than females (i.e., LM < LF in case of negative values; Figure 4B and Table 1). For the vmPFC(−) networks, males showed stronger strength of connectivity with the dorsolateral PFC, SMG, and SMA, all in the right hemisphere, compared to females (i.e., LM < LF in case of negative values; Figure 5 and Table 1).

Interestingly, like pMCC(−) network that showed gender differences connected to cerebellum in Crus I, left pINS(−) network and left AMYG(+) network that showed gender-related differences also connected to cerebellum but in Crus I and VI/Crus I, respectively (i.e., LM < LF in case of negative values for connectivity between pINS and Crus I; LF > LM for connectivity between left AMYG and VI/Crus I, respectively; Supplementary Figure S2 and Table 1). There were no gender differences for the other seeded-networks in the late-adulthood stage.

### Age-Related Differences in Females and Males

**FIGURE 6:**
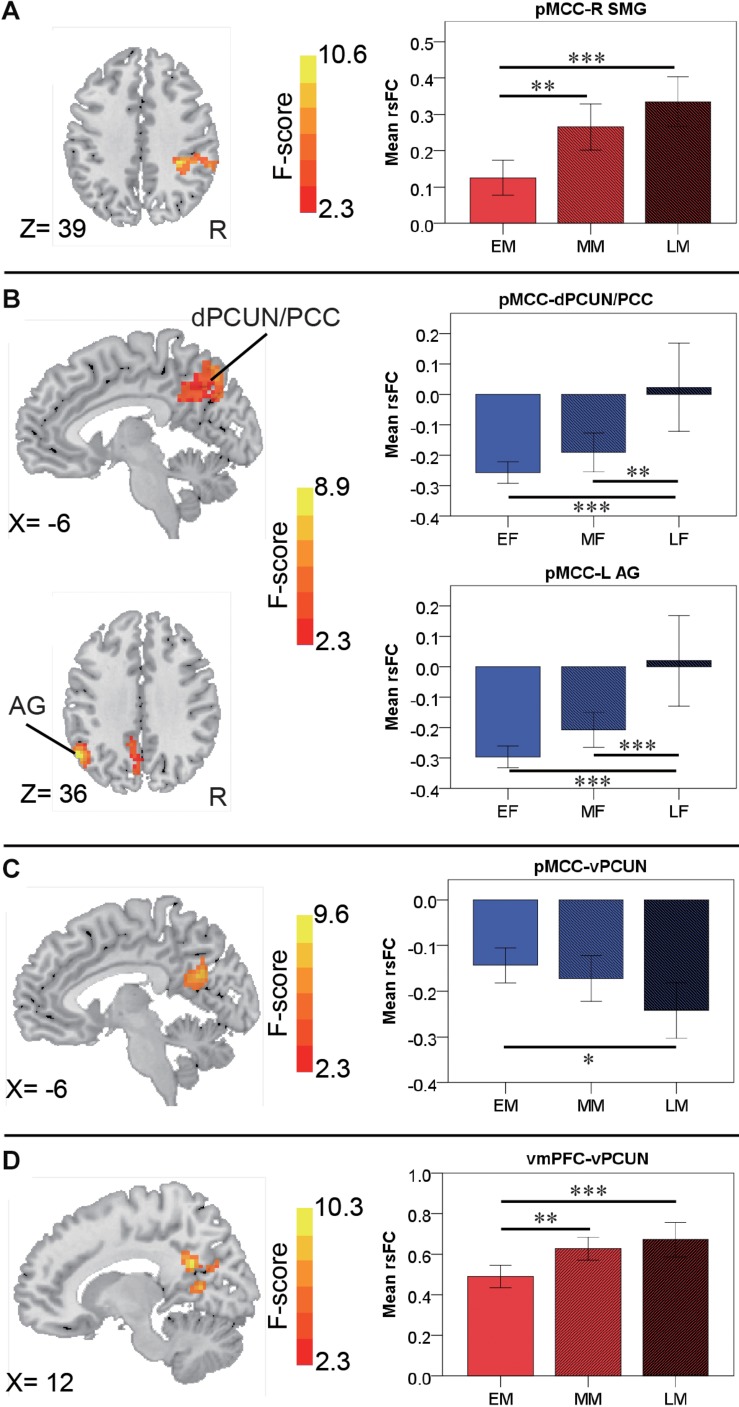
Regions showing the age effect identified through ANCOVA (with a permutation test correction threshold of *p* < 0.05, and *post hoc* comparisons) in the pMCC(+) network in the males **(A)**, in the pMCC(–) network in the females **(B)** and in the males **(C)**, and in the vmPFC(+) network in the males **(D)**, each with the corresponding mean strength between the seed and region for the three different stages shown in the bar plot, error bars indicated two standard errors, and asterisks indicated significant differences with Bonferroni correction (^∗^*p* < 0.05, ^∗∗^*p* < 0.01, ^∗∗∗^*p* < 0.001; pMCC, posterior midcingulate cortex; vmPFC, ventromedial prefrontal cortex; SMG, supramarginal gyrus; dPCUN, dorsal precuneus; PCC, posterior cingulate cortex; AG, angular gyrus; vPCUN, ventral precuneus; L, left; R, right).

For the pMCC(+) network, the effect of age was not significant in females. By contrast, in males, the effect of age was detected in the right SMG, with the strength of the connectivity increasing as a function of age (i.e., LM > MM > EM; Figure 6A and Table 2). *Post hoc* analyses indicated the significantly stronger strength in both LM and MM compared to EM.

**TABLE 2:** Regions showing the age effect on the functional connectivity for the pMCC networks in the females and males.

**Region (BA)**		**MNI coordinates**			**Peak score (*F*-score)**	**Cluster size (voxels)**
		***x***	***y***	***z***		
**Female**						
pMCC(−): dPCUN/PCC (7/31)	B	–6	–72	48	7.04	204
pMCC(−): AG (39)	L	–54	–60	36	8.94	178
**Male**						
pMCC(+): SMG (40)	R	33	–36	39	10.60	190
pMCC(−): vPCUN (31)	B	–12	–63	21	9.58	182
vmPFC(+): vPCUN (31)	B	12	–54	24	10.25	223

*Thresholds were set at *p* < 0.05, permutation test corrected. BA, Brodmann area; MNI, Montreal Neurological Institute; pMCC, posterior midcingulate cortex; vmPFC, ventromedial prefrontal cortex; dPCUN, dorsal precuneus; AG, angular gyrus; SMG, supramarginal gyrus; vPCUN, ventral precuneus; L, left; R, right; B, bilateral.*

For the pMCC(−) network, in females, the ANCOVA detected two clusters in the dorsal PCUN and left AG, both with the strength of the corresponding rsFC increasing with age (i.e., EF < MF < LF in case of negative values; Figure 6B and Table 2). *Post hoc* analyses confirmed that both EF and MF participants showed significantly stronger strength than LF. In males, the ventral PCUN negatively correlated with the pMCC, with the connectivity becoming stronger as a function of age (i.e., LM < MM < EM in case of negative values; Figure 6C and Table 2); *post hoc* analyses indicated significant differences between LM and EM.

For the vmPFC(+) network, in males, the effect of age was detected in the ventral PCUN, with the strength of the connectivity increasing with age (i.e., LM > MM > EM; Figure 6D and Table 2). *Post hoc* analyses indicated that both LM and MM showed a significantly stronger strength than EM. The effect of age was not significant in the connectivity from the other three seeds, both in females and males.

## Discussion

In the present study, we systematically investigated the gender- and age-specific differences in the rsFC of the CAN in adulthood with five functionally predefined core regions, namely pMCC, left AMYG, right aINS, left pINS, and vmPFC using the larger cross-sectional adulthood sample from the Southwest University adult lifespan database (Wei et al., 2018). The results are interpreted in the characteristics of the seed associated with autonomic outflow (i.e., sympathetic and parasympathetic regulation) and large-scale brain networks (Yeo et al., 2011; Beissner et al., 2013). Overall, the positive and negative rsFC maps from each seed in different stages of female and male adults were strongly coupled with one of the large-scale brain networks such as sensorimotor, attentional, basal ganglia, limbic networks, and DMN. This results demonstrated that the autonomic responses can be also represented intrinsically in the resting brain, as they have been previously found to engage across somatosensory-motor, attention, and affective tasks (Beissner et al., 2013). Importantly, connectivity seeded from pMCC and vmPFC revealed gender-related differences selectively in the early- and late-adulthood stages, and they also changed as a function of age selectively in females and males. Moreover, connectivity seeded from right aINS demonstrated gender-related differences in the early-adulthood stage.

The seeds of pMCC and left pINS showed similar pattern of positive correlations with sensorimotor and basal ganglia networks, which is consistent with previous findings (Taylor et al., 2009; Cauda et al., 2011; Deen et al., 2011; Sie et al., 2019). These involved areas have been identified for maintaining optimal physiological conditions in the body and responding to both interior and exterior environmental challenges, ranging from exercise, altitude sickness, and social interactions (Craig, 2002; Saper, 2002; Critchley, 2004). Moreover, the right aINS(+) network was involved in attentional and basal ganglia networks, which is consistent with previous studies reporting its role in reorienting attention in response to baroreceptor unloading, handgrip exercise, gut, and acupuncture stimulation (Kimmerly et al., 2005; Wong et al., 2007a; Suzuki et al., 2009; Maihofner et al., 2011). These aforementioned connectivities of the cingulate cortex (i.e., pMCC) and insula (i.e., left pINS and right aINS) with basal ganglia support the visceral afferent information in the interoceptive pathways that work as critical connectors in motor control to facilitate body movement (Craig, 2002; Critchley and Harrison, 2013; Critchley and Garfinkel, 2015).

On the other hand, the seeds of pMCC and right aINS showed a similar pattern of negative correlations with the DMN. Their cortical activation has been associated with muscle contraction, pain, and acupuncture stimulation, which is also correlated with deactivation in the DMN (Goswami et al., 2011; Maihofner et al., 2011; Napadow et al., 2013). Collectively, the pMCC-, right aINS-, and left pINS-seeded networks found in our study correspond to the concept of posterior (e.g., pMCC and pINS) and anterior (e.g., anterior MCC and aINS) functional systems that integrate multiple cognitive, homeostatic, and emotional (interoceptive) functions (Craig, 2002, 2009); and we further provide evidence of their connectivity at the resting state (Sie et al., 2019).

For the gender-related differences in the early-adulthood stage, the negative rsFC of the pMCC and right aINS with the ventral PCUN/PCC, and of pMCC with the right vmPFC were both stronger in females as compared to males. The ventral PCUN/PCC has been reported to engage in internally focused tasks including autobiographical memory retrieval, envisioning the future, and conceiving the perspectives of others (Cavanna and Trimble, 2006; Zhang and Li, 2012). Recalling the previous experience associated with peripheral physiological response produces a coordinated set of behavioral, autonomic, and metabolic changes that promote an adaptive response to environmental demands (Wager et al., 2009). Moreover, the decreased activity in the right vmPFC (and also the right putamen) has been found to couple with increased heart rate elicited by social evaluative threat in the public speech preparation tasks (Wager et al., 2009). More directly related to our findings, the premenopausal women tended to evoke stronger vmPFC and ventral PCUN/PCC deactivation and weaker right INS and dorsal ACC activations related to weaker sympathetic regulation than men at the same age (i.e., lower heart rate and mean arterial pressure responses to handgrip exercise) (Wong et al., 2007a). The authors suggested that sex hormonal estrogen might influence the magnitude of the peripheral response in cardiovascular control and the cortical activation pattern (Wong et al., 2007a). Based on these findings, the stronger connectivity from pMCC and right aINS to ventral PCUN/PCC, as well as from pMCC to right vmPFC in (premenopausal) females compared to males might possibly reflect the influence of estrogen to suppress the sympathoexcitation in the central autonomic regulation (Kimmerly et al., 2007; Wong et al., 2007a; Macey et al., 2016). In this study, the disappearance of the gender-related differences in these connections in the middle-adulthood stage might be related to the gradually decreased estrogen in midlife females (Kuo et al., 1999; Liu et al., 2003).

We further found gender-related differences of connectivity seeded from the pMCC specifically in the late-adulthood stage. The results showed that elderly females (i.e., LF) had a stronger positive connection to the right postcentral gyrus, and a weaker negative connection to the PCUN, left AG, left middle temporal visual area, and bilateral frontal eye field than elderly males (i.e., LM). The postcentral gyrus receives all sensory inputs from the body including the touch and pain (Maihofner et al., 2011; Napadow et al., 2013). The PCC, PCUN, and middle temporal visual area (and also in the bilateral vmPFC, orbitofrontal cortex, and cuneus) have their deactivation positively correlated with sympathetic vasoconstrictor responses also to touch and pain stimulation (Maihofner et al., 2011). In the same vein, a robust deactivation in the DMN is correlated with heart rate deceleration to specific acupuncture stimulation (Napadow et al., 2013). On the other hand, higher (but not lower) activity in the left AG during resting state was found in fibromyalgia patients when their spontaneous pain was reduced by music (Garza-Villarreal et al., 2015). Higher activity in the postcentral gyrus (and also anterior MCC, right anterior/mid INS, and SMG) was also found in patients with Alzheimer’s disease compared to healthy controls when receiving pain (Cole et al., 2006). More generally, patients with mild cognitive impairment and Alzheimer’s disease showed altered DMN including the PCC, AG, and frontal eye field (and also HF) (Jones et al., 2011; Wang et al., 2011). Based on the findings of these studies, even they did not investigate gender differences and some even having opposite pattern with our results, we interpret the gender differences of connectivity seeded from pMCC found in the late-adulthood as possible evidence of autonomic regulation specifically for pain and they might be related to Alzheimer’s disease in the elderly. Future studies are needed to validate this speculation.

Regarding the age-related differences in adults, there was a significant effect of age in females’ connectivity from pMCC to the dorsal PCUN/PCC and left AG, and the strength of connectivity decreased with advancing age. The dorsal PCUN/PCC has been identified as a prominent role in parasympathetic regulation for somatosensory-motor tasks (Beissner et al., 2013). By using resting-state fMRI, the activity of dorsal PCUN/PCC and left AG is found negatively correlated with the externally oriented system in auditory, somatosensory, and visual cortices (Tian et al., 2007). Moreover, as mentioned previously, activity in the left AG during resting state was related to pain experience (Garza-Villarreal et al., 2015). Although not being directly related, we suspect the stronger connections found in the EF and MF than LF might reflect their stronger autonomic regulation for menstrual pain before menopause. On the contrary, the effect of age found in males was demonstrated by positive connectivity of pMCC with right SMG, and its strength increased as a function of age. The cooperation between these two areas has been found to engage in perception of space and limbs location for fine motor control and motor skill acquisition (Vogt, 2016). The right SMG also plays a key role in proprioception referring to knowledge of the spatial location of one’s limb in the absence of vision and the right lateral proprioception-related brain activation was reduced in the stroke participants (Ben-Shabat et al., 2015). Moreover, the activity of pMCC and SMG have been associated with peripheral sympathetic arousal, for instance, due to mental stress (psychogenic) or increased body temperature (thermogenic) (Beissner et al., 2013; Farrell et al., 2015). Although decreased rsFC due to aging is a common pattern reported in resting-state fMRI studies, the connectivity within the sensorimotor network has been found to become stronger in normal aging (Geerligs et al., 2015). Based on these findings, we interpret the stronger connectivity strength in the sensorimotor network as advancing age in males as a greater recruitment of sympathetic CAN subdivisions to maintain the reflexive movement with elevated autonomic stress in the middle- and late-adulthood males.

Furthermore, the vmPFC was positively correlated with the DMN, basal ganglia and limbic networks, and negatively correlated with the attentional network. These maps nicely represent the cortical substrates associated with cardio-autonomic function during baroreceptor unloading (Kimmerly et al., 2005; Kimmerly et al., 2007), handgrip exercise (Wong et al., 2007a, b; Napadow et al., 2008), public speech preparation (Wager et al., 2009), noxious stimulation (Maihofner et al., 2011; Beissner et al., 2012), and acupuncture stimulation events (Napadow et al., 2013). Specifically, the vmPFC is related to a default baseline homeostatic state of brain function in a physiological account of efferent vagal activity associated with tonic reduction in autonomic arousal (Nagai et al., 2004b; Zhang et al., 2014). Therefore, the connectivity of the vmPFC found in our study provides evidence of an intrinsic link between cardio-autonomic function and selective attention.

The vmPFC-seeded negative connectivity had gender-related differences with lower activity in the elderly females (i.e., LF), as connected to the right dorsolateral PFC, SMG, and SMA. The activity of right dorsolateral PFC and right SMG (also the right frontal eye field, rostral ACC) has been found to be positively correlated with the high frequency component of heart rate variability due to emotion (Lane et al., 2009). We thus suggest that the weaker connectivity in females may shed some light in the context of central autonomic processing related to attention and emotion, and it might possibly account for the higher prevalence of anxiety and depression disorder in elderly females compared to males at the same age (Girgus et al., 2017). Intriguingly, we also found that an increased connectivity of vmPFC with vPCUN/PCC as advancing age in males. The two areas are considered as important components of efferent vagal activity to the heart (Ziegler et al., 2009), we thus interpret this stronger connectivity along age associated with the greater parasympathetic regulation in middle-aged and elder males. These opposite patterns between the sympathetic (i.e., pMCC to right SMG) and parasympathetic connections (i.e., vmPFC to ventral PCUN/PCC) might further reflect an increased vagal antagonism of sympathetic influences due to aging in cardio-autonomic function, particularly in males.

## Conclusion and Limitations

The seeds used in this study have been reported to engage in autonomic regulation engaging various cognitive tasks, we further investigated their connectivity during the resting state. Results revealed their correlations with the large-scale brain networks, and they also substantiated the findings in literature by showing the anti-correlations between the sympathetic and parasympathetic regions, which might connect the important subsystems for the central autonomic processing. For the gender-related differences in the early-adulthood stage, females had stronger negative rsFC of pMCC and right aINS with the medial DMN, possibly reflecting the greater suppression of the sympathoexcitation in the CAN. Moreover, specifically in the late-adulthood stage, females showed prominent decline in the anti-correlations with the most DMN and right ventral attentional network than males. Such gender differences might relate to the risk of dementia, depression, and anxiety in the elderly, which may serve as a biomarker relating to the healthy longevity and vitality in late life span. Moreover, females demonstrated reduced negative rsFC from the pMCC to the dorsal PCUN/PCC and left AG, whereas males showed the opposite pattern, namely an increased connectivity, between pMCC and right SMG, and between vmPFC and ventral PCUN with advancing age. These results imply their changes in the central autonomic processing associated with pain experience and reflective movement, respectively, due to normal aging. In sum, the gender- and age-related differences could be detected at neural level, as represented in the rsFC, and they might be associated with sex hormones and sensorimotor abilities.

There were some other limitations in our present work. First, the database did not include any psychophysiology tests to score physiological states or neuropsychological performance. Future studies combined with such assessments would clarify the differentiation associating central autonomic processing in body–brain–mind axis. Second, in the conventional fMRI, there is a technical limit about temporal resolution at a TR of 2,000 ms and the aliasing effects from cardiac and respiratory fluctuations due to the change of carbon dioxide concentration in the venous vasculature, especially in the AMYG (Boubela et al., 2015). Future studies using short TR sequence with multiband sequences and/or combining with structural connectivity are suggested to better understand the mechanism associating with central autonomic processing. Third, we found gender-related differences in the elderly-adulthood stage with the connectivity seeded from pMCC, left AMYG, and left pINS to the cerebellum crus I and/or VI. Functional connectivity of the cerebellum crus I and VI is identified as area associated with autonomic regulation in the meta-analysis study (Beissner et al., 2013). However, most functional images in the current database covered half of the cerebellum, which could not provide full spectrum of cerebellum. Finally, the gender- and age-related CAN changes in this study were found using cross-sectional data from college students and staff at the university and therefore could be influenced by potential cohort effects. The longitudinal network dynamics need to be examined to confirm our current findings.

## Data Availability Statement

Publicly available datasets were analyzed in this study. These data can be found here: http://fcon_1000. projects.nitrc.org/indi/retro/sald.html.

## Ethics Statement

The data project was approved by the Research Ethics Committee of the Brain Imaging Center of Southwest University with written informed consent from all subjects prior to the study.

## Author Contributions

Y-HS and W-CC conceived and designed the study, and supervised the project. J-HS analyzed the data. J-HS and Y-HC interpreted the data and wrote the manuscript. All authors edited the manuscript.

## Conflict of Interest

The authors declare that the research was conducted in the absence of any commercial or financial relationships that could be construed as a potential conflict of interest.

## Footnotes

1 http://www.fil.ion.ucl.ac.uk/spm
