# Supplementary material for: Gender- and Age-Specific Differences in Resting-State Functional Connectivity of the Central Autonomic Network in Adulthood
Source: Front Hum Neurosci. 2019 Oct 17;13:369. doi: 10.3389/fnhum.2019.00369 (PMC6811649; doi:10.3389/fnhum.2019.00369)
Supplement: Supplementary file 1 [file Data_Sheet_1.docx]

**RESULTS**

**Demographic Characteristics of Participants**

**TABLE S1 |** Demographic data of females

| **Variable** | **EF** | **MF** | **LF** | **ANOVA** | ***p*-value^a^** |  |  |
| --- | --- | --- | --- | --- | --- | --- | --- |
|  | **N = 103** | **N = 125** | **N = 65** |  | **EF vs MF** | **EF vs LF** | **MF vs LF** |
| Age | 24.5 ± 3.5 | 50.1 ± 6.5 | 66.5 ± 5.8 | <0.001 | <0.001 | <0.001 | <0.001 |
| Mean FD | 0.14 ± 0.07 | 0.19 ± 0.11 | 0.24 ± 0.13 | <0.001 | <0.001 | <0.001 | 0.008 |
| GMV | 691 ± 43.6 | 616 ± 56.7 | 564 ± 45.3 | <0.001 | <0.001 | <0.001 | <0.001 |
| WMV | 445 ± 40.6 | 447 ± 50.7 | 425 ± 45.3 | 0.005 | 1.000 | 0.024 | 0.005 |
| CSF | 293 ± 46.8 | 348 ± 66.6 | 380 ± 73.4 | <0.001 | <0.001 | <0.001 | 0.002 |

Data presented as mean ± SD. EF, early-adulthood females; MF, middle-adulthood females; LF, late-adulthood females; N: sample size; ANOVA, analysis of variance; FD, frame-wise displacement; GMV, gray matter volume; WMV, white matter volume; CSF, cerebrospinal fluid. ^a^ *p* value was obtained by post-hoc comparison with Bonferroni correction.

**TABLE S2 |** Demographic data of males

| **Variable** | **EM** | **MM** | **LM** | **ANOVA** | ***p*-value^a^** |  |  |
| --- | --- | --- | --- | --- | --- | --- | --- |
|  | **N = 67** | **N = 60** | **N = 47** |  | **EM vs MM** | **EM vs LM** | **MM vs LM** |
| Age | 25.3 ± 3.7 | 50.4 ± 7.3 | 66.2 ± 5.7 | <0.001 | <0.001 | <0.001 | <0.001 |
| Mean FD | 0.13 ± 0.06 | 0.18 ± 0.09 | 0.25 ± 0.13 | <0.001 | 0.006 | <0.001 | 0.001 |
| GMV | 744 ± 56.9 | 646 ± 54.1 | 610 ± 55.7 | <0.001 | <0.001 | <0.001 | 0.003 |
| WMV | 482 ± 39.9 | 474 ± 41.5 | 464 ± 46.4 | 0.088 | 0.897 | 0.083 | 0.673 |
| CSF | 347 ± 59.3 | 363 ± 59.0 | 433 ± 85.7 | <0.001 | 0.500 | <0.001 | <0.001 |

Data presented as mean ± SD. EM, early-adulthood males; MM, middle-adulthood males; LM, late-adulthood males; N: sample size; ANOVA, analysis of variance; FD, frame-wise displacement; GMV, gray matter volume; WMV, white matter volume; CSF, cerebrospinal fluid. ^a^ *p* value was obtained by post-hoc comparison with Bonferroni correction.

**
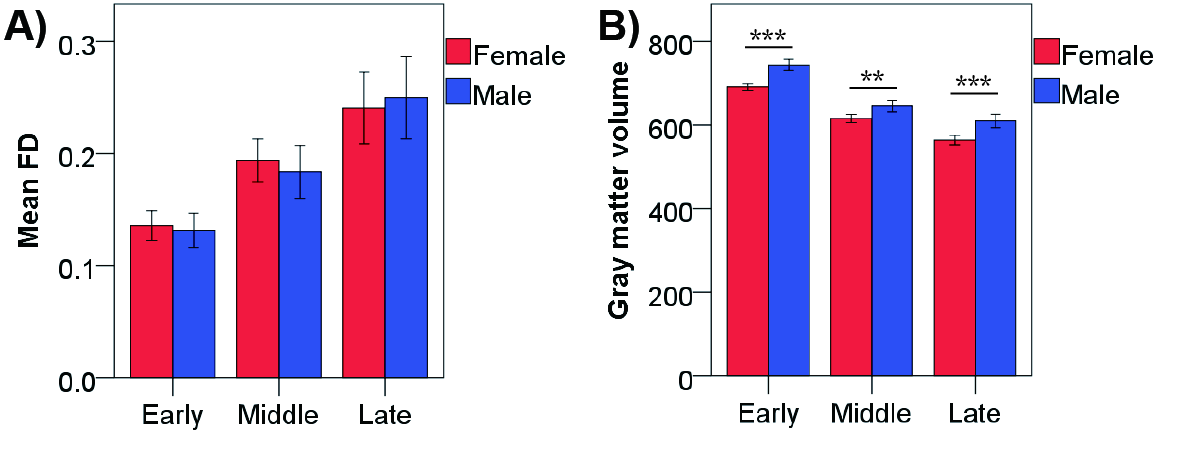
**

**FIGURE S1 |** Gender differences of the head motion (i.e., FD) **(A)** and gray matter volume **(B)** in early-, middle- and late-adulthood stages identified by separate two-sample *t* tests. Error bars indicated two standard errors and asterisks indicated significant differences (** *p* < 0.01, *** *p* < 0.001).


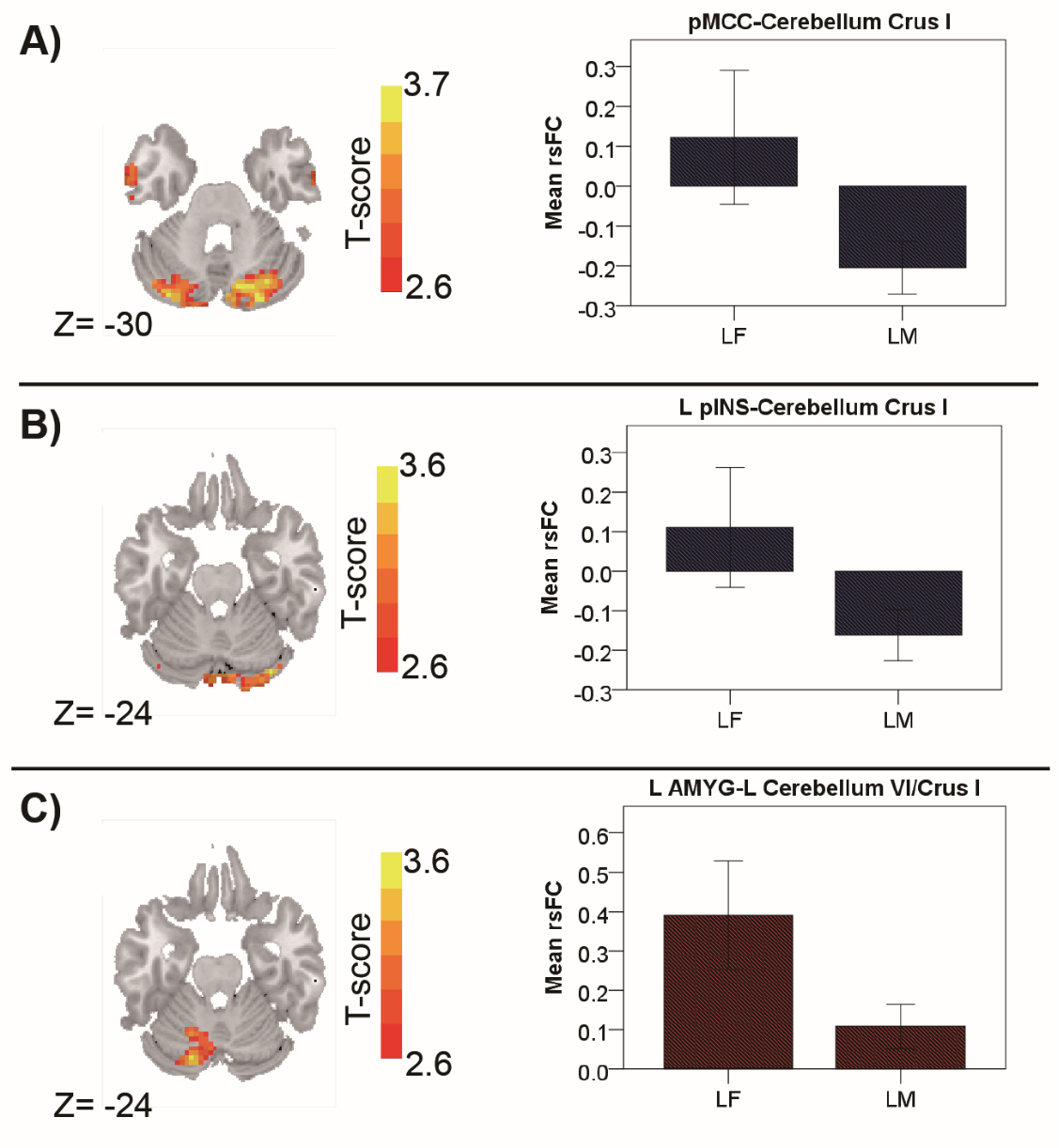


**FIGURE S2 |** Regions showing the gender differences in the late-adulthood stage detected by two-sample *t* test (with a permutation test correction threshold of *p* < 0.05) in the pMCC(−) network **(A)**, the left pINS(−) network **(B)**, and the left AMYG(+) network **(C)**, each with the corresponding mean strength between the seed and region for males and females shown in the bar plot, error bars indicated two standard errors (pMCC, posterior midcingulate cortex; pINS, posterior insula; AMYG, amygdala; L, Left).
